# Knowledge, attitudes and practices of COVID-19 among income-poor households in the Philippines: A cross-sectional study

**DOI:** 10.7189/jogh.10.011007

**Published:** 2020-06

**Authors:** Lincoln Leehang Lau, Natalee Hung, Daryn Joy Go, Jansel Ferma, Mia Choi, Warren Dodd, Xiaolin Wei

**Affiliations:** 1 International Care Ministries Inc., Manila, Philippines; 2 School of Public Health & Health Systems, University of Waterloo, Waterloo, Canada; 3 Dalla Lana School of Public Health, University of Toronto, Toronto, Canada

## Abstract

**Background:**

The presence of COVID-19 in low- and middle-income countries (LMICs) is raising important concerns about effective pandemic response and preparedness in the context of fragile health systems and the pervasiveness of misinformation. The objective of this study was to gain an understanding of how COVID-19 was perceived by households experiencing extreme poverty in the Philippines.

**Methods:**

This study was conducted in partnership with International Care Ministries (ICM), a Philippine-based non-governmental organization (NGO) that runs a poverty-alleviation program called *Transform* targeted towards extreme low-income households. We integrated knowledge, attitudes, and practices (KAP) questions into ICM’s cross-sectional program monitoring and evaluation systems from February 20 through March 13, 2020. Frequencies and proportions were calculated to describe the respondents’ responses, and the Kruskal-Wallis test and binomial logistic regression were undertaken to determine the socio-demographic characteristics associated with COVID-19 KAPs.

**Results:**

In total, 2224 respondents from 166 communities in rural, urban and coastal settings were surveyed. Although the survey was administered during the earlier stages of the pandemic, 94.0% of respondents had already heard of COVID-19. Traditional media sources such as television (85.5%) and radio (56.1%) were reported as the main sources of information about the virus. Coughing and sneezing were identified as a transmission route by 89.5% of respondents, while indirect hand contact was the least commonly identified transmission route, recognized by 72.6% of respondents. Handwashing was identified by 82.2% of respondents as a preventive measure against the virus, but social distancing and avoiding crowds were only identified by 32.4% and 40.6%, respectively. Handwashing was the most common preventive practice in response to COVID-19, adopted by 89.9% of respondents. A greater number of preventive measures were taken by those with more knowledge of potential transmission routes.

**Conclusions:**

There is a need for targeted health education as a response strategy to COVID-19 in low-income settings, and it is important that strategies are contextually relevant. Understanding KAPs among populations experiencing extreme poverty will be important as tailored guidance for public health response and communication strategies are developed for LMICs.

The presence of the COVID-19 pandemic in low- and middle-income countries (LMICs) is raising important concerns about the preparedness of health systems within these countries to address the disease as it continues to spread. With health care facilities that were already overburdened before the pandemic, it is becoming increasingly clear that adopting the measures employed by high-income countries in LMICs may not be feasible [1,2]. Current recommendations focus heavily on hospital-based interventions, but in the context of severe resource limitations, addressing shortages of hospital beds, oxygen, ventilators and personal protective equipment as primary response initiatives may not be realistic. Moreover, there is a need within LMICs to provide emergency support to vulnerable populations, including individuals and households experiencing poverty.

There are also concerns around misinformation that may impede public health responses. As the WHO Director-General Dr Tedros Adhanom Ghebreyesus said, “we’re not just fighting an epidemic; we’re fighting an infodemic” [3]. The reach of the pandemic on a global scale has led to a flood of information surrounding the virus, and despite the merits of rapid information dissemination through mass and social media for public health action, misinformation can also easily be propagated through the same channels [4-6]. Both exaggerated and understated pandemic estimates [7] can either fuel panic or a false sense of security among the general public. Additionally, confusion surrounding basic information on how to reduce transmission and exposure to the virus puts people at risk of infection [8,9]. In the context of LMICs, households in resource-poor settings might not have access to regular and reliable sources of information about disease etiology, leaving them ill-equipped to minimize the risk of infection during emerging outbreaks [10-12]. Understanding public perceptions and their responses to COVID-19 is therefore critical in the ongoing planning and implementation of effective pandemic responses in LMICs, particularly by evaluating current public health messaging and communication strategies.

In the Philippines, although the speed of transmission was initially limited, public health measures have not been sufficient to curtail the spread of the virus. On January 30, 2020, its Department of Health confirmed the first case in the country [13], and in an attempt to contain further transmission, the Philippine government enforced sweeping preventive measures such as an enhanced community quarantine in regions with significant numbers of COVID-19 cases [14]. These measures involved imposing strict home quarantine, implementing lockdowns in places with positive COVID-19 cases, suspending public transportation systems, and restricting air and sea travel [15]. However, as of April 27, 2020, 7579 cases of COVID-19 have been confirmed with numbers continuing to exponentially increase [16]. Although the caseload has mostly been concentrated in the country’s capital region of Metro Manila, there is evidence of local transmission prior to the spike in cases, and the likely spread to surrounding regions threaten communities that have limited health system capacity [17].

The objective of this study was to understand the knowledge, attitudes, and practices (KAP) of COVID-19 among households experiencing extreme poverty in the Philippines. A number of studies examining KAPs on COVID-19 have already been conducted [18-24], but there is a need to understand KAPs among vulnerable and low-income communities. As various guidelines and strategies are developed to address COVID-19 in LMICs, it is important that households from lower socioeconomic positions are equitably included in these initiatives.

## METHODS

### Study design

This study was conducted in partnership with International Care Ministries (ICM), a Philippine-based non-governmental organization (NGO) that runs a poverty-alleviation program called *Transform* targeted towards extreme low-income households. ICM regularly surveys a random proportion of *Transform* program participants for monitoring and evaluation purposes, and these surveys have been utilized for health research in the past [25]. These surveys are face to face interviews, approximately 30-45 minutes in length, and are conducted in local languages (Tagalog, Bisaya, and Hiligaynon) by trained surveyors who would travel to each respondent’s residence.

We integrated 16 KAP questions related to COVID-19 into ICM’s program monitoring and evaluation systems from February 20 through March 13, 2020. These questions were based on KAP survey instruments from previous studies of the 2009 H1N1 pandemic in China [26] and the SARS epidemic in Hong Kong [27]. The questions covered the three KAP domains: 1) eight knowledge-based questions determined respondents’ awareness of the virus, its main modes of transmission, and preventive measures; 2) two attitude-based questions assessed the participants’ response to virus-related symptoms and their COVID-19 risk perceptions; and 3) six practice-based questions determined the general preventive measures respondents had taken in response to the new virus. Respondents were also asked about how they received their information about COVID-19 (see Appendix S1 in the **Online Supplementary Document** for the full list of questions).

The surveys were designed specifically for participants of ICM’s poverty alleviation program. As a result, all respondents were pre-screened by ICM and partnering community leaders and included if identified to be experiencing extreme poverty based on a scorecard measure that included self-reported income and physical dwelling characteristics. The KAP questions were added to a pre-scheduled monitoring and evaluation survey for a subset of 2428 *Transform* participants from 166 communities. The communities represented 32% of *Transform* participants who had been interviewed prior to the program intervention in October 2019. The surveys covered households in rural, urban, and coastal settings from the following provinces in the Philippines: Palawan, Aklan, Roxas, Iloilo, Negros Occidental, Negros Oriental, Bohol, Zamboanga Del Norte, and South Cotabato. Collection of data was approved by the University of Toronto Research Ethics Board (Protocol Number: 39138). All respondents provided verbal informed consent prior to the collection of any data with the knowledge they could refuse to answer any question, withdraw from the survey at any point, and that all data would remain confidential. All survey data were collected on mobile devices using SurveyCTO, a computer-aided personal interviewing (CAPI) platform which collects responses via an app and stores data in a secure server. CAPI approaches have important advantages with the reduction of missing data [28] and SurveyCTO has also been used in other research conducted in lower- and middle-income contexts [29,30].

### Statistical approach

Frequencies and proportions were first calculated to describe the respondents’ knowledge, attitudinal responses, and preventive measures taken against COVID-19, as well as the sources they consulted for information about the virus.

The knowledge- and practice-based questions were summed to create an index for each that were used as continuous variables for analysis. As these continuous variables were not normally distributed, the Kruskal-Wallis nonparametric test was used to determine the socio-demographic characteristics associated with COVID-19 knowledge and practices, including income level, geographical location, educational attainment, access to mobile phones or televisions, and social ties with regional health and government units. The test identified whether the categories within each socio-demographic variable significantly differed on each outcome. The respective mean and confidence intervals of the knowledge and practices variables were then calculated for each categorical level to further describe the differences.

The attitude-based question about respondents’ risk perception of COVID-19 was a binary variable that indicated whether or not a respondent was worried about contracting the virus. Multivariate logistic regression analysis was applied to investigate socio-demographic factors that may be associated with risk perception. Additional regression analyses were performed to determine the impact of select knowledge and attitudes indicators on the respondents’ risk perception and preventive practices adopted in light of COVID-19. Mean estimates and odds ratios, with their corresponding 95% confidence intervals (CIs), were obtained from the Kruskal-Wallis tests and logistic regression analyses, respectively. Appropriate pre-estimation procedures, including calculating the variance inflation factor and robust standard errors, were performed to ensure the absence of multicollinearity and heteroskedasticity in the logistic regression models. Individuals who did not indicate that they had heard of COVID-19 were not asked to complete the subsequent KAP questions, and cases with missing data were excluded from the logistic analyses.

All statistical analyses were conducted using STATA 13 (StataCorp, College Station, TX, USA) and R version 3.2.3 (R Core Team, R Foundation for Statistical Computing, Vienna, Austria).

## RESULTS

### Respondent characteristics

A total of 2428 participants in the *Transform* program representing 166 communities were targeted to be surveyed. Of these, we successfully collected questionnaires from 2224 participants (91.6%). The remaining 204 individuals did not provide consent to participate in the survey. Background characteristics of the survey respondents are presented in **Table 1**. The average age was 41.3 years old (SD = 14.6), and 2061 (93%) were female. Among the respondents, 1795 (80.8%) had not graduated from secondary school, and 977 (44%) had only completed elementary education or less. The average household self-reported a monthly income of 7657 Philippine Pesos (PHP) (US$151.26) (standard deviation (SD) = PHP8062 or US$ 159.26), which places the average household in this study in the bottom 5th percentile of the country [31]. There were 1725 (78.1%) respondents that reported living in rural areas with the remaining 283 (12.8%) and 200 (9.1%) participants residing in coastal and urban areas, respectively. The predominant industry differs in each of these geographical areas, and they also describe the density and relative proximity of households within communities (Table S1 in the **Online Supplementary Document**). Among all households surveyed, 1773 (79.7%) had electricity, 1084 (48.7%) owned a working television, and 1733 (77.9%) of respondents reported owning at least one mobile phone. 1391 (62.6%) respondents were also covered by PhilHealth, the government body that administers the National Health Insurance Program in the Philippines.

**Table 1:** Characteristics of survey respondents

Characteristic	No.	%
**Sex:**		
Female	2061	92.7
Male	163	7.3
**Age (in years):**		
<20	50	2.2
20-39	1080	48.6
40-59	783	35.2
60	311	14.0
**Education:**		
No education	48	2.2
Elementary	929	41.8
Partial high school	818	36.8
High school graduate or ALS	213	9.6
Partial college or vocational school	165	7.4
Complete college or higher	51	2.3
**Income per person per day (self-reported):**		
Less than US$ 1.00	1262	56.8
US$ 1.00-US$ 2.00	710	32.0
Greater than US$ 2.00	248	11.2
**Electricity:**		
Yes	1773	79.7
No	451	20.3
**Number of mobile phones:**		
None	491	22.1
One	1064	47.8
Two	480	21.6
Three or more	189	8.5
**Television:**		
Yes, and it is in working condition	1084	48.7
Yes, but it is out of order or not working anymore	116	5.2
No	1024	46.0
**Geotype:**		
Coastal	283	12.8
Rural	1725	78.1
Urban	200	9.1
**Has national health insurance (PhilHealth):**		
Yes	1391	62.6
No	824	37.1
I don't know	7	0.3

ALS – alternative learning system

### Information sources for COVID-19

Of the 2090 respondents who indicated they were aware of the virus, 1786 (85.5%) and 1173 (56.1%) reported learning and staying up to date about COVID-19 through television and radio, respectively (**Table 2**). There were 908 (43.4%) people that reported obtaining information from friends, family or neighbours. By contrast, only 230 (11.0%) and 432 (20.7%) people reported consulting internet or social media sources, respectively, for information about COVID-19.

**Table 2:** Information sources for COVID-19 reported by survey respondents

Question (n = 2090)	n	%
**Where did you learn and stay up to date about COVID-19?:**		
Television	1786	85.5
Radio	1173	56.1
Social media (Facebook, Instagram, etc.)	432	20.7
Internet (websites, blogs, etc.)	230	11.0
Friends, relatives, and/or neighbours	908	43.4
Local government officials	479	22.9
Announcements at work	51	2.4
Other	10	0.5

### COVID-19 knowledge

**Figure 1:**
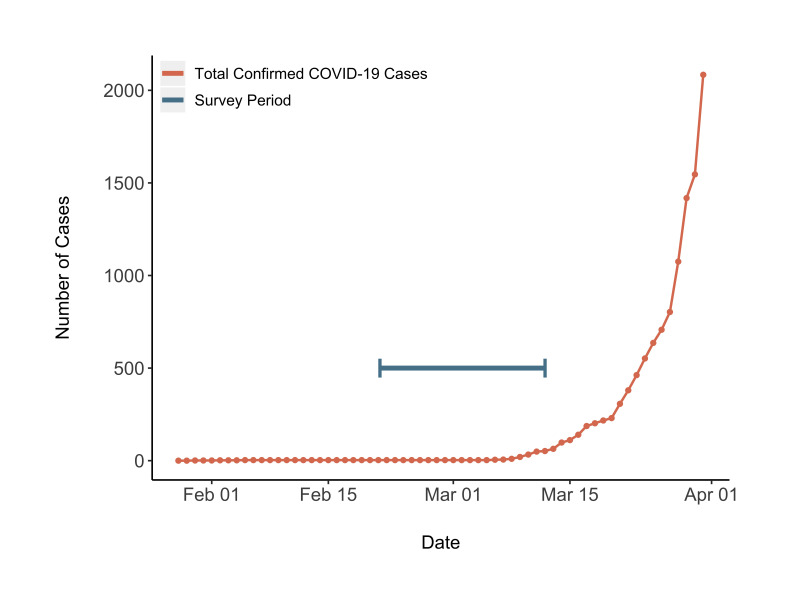
Confirmed COVID-19 cases over time in the Philippines and International Care Ministries (ICM) survey period.

The survey was conducted prior to the wider spread of COVID-19 in the Philippines (**Figure 1**) [32], but 2090 (94.0%) respondents had already heard of this novel virus by this time (**Table 3**). Only the respondents who indicated they were aware of the virus were asked to complete the subsequent KAP questions. Of the 2090 respondents, 1870 (89.5%) were able to identify coughing and sneezing as a transmission route, while the least commonly recognized transmission route was indirect hand contact, identified by 1518 respondents (72.6%).

**Table 3:** Knowledge, attitudes and practices toward COVID-19 among income-poor households in the Philippines

Questions (N=2090)	Yes (%)	No (%)	Don't know (%)
**Knowledge**			
Have you heard of the new coronavirus (COVID-19)?*	2090 (94.0)	117 (5.2)	17 (0.8)
Can COVID-19 be transmitted (caught or spread) by:			
Coughing and sneezing?	1870 (89.5)	116 (5.5)	104 (5.0)
Face to face talking?	1735 (83.0)	206 (9.9)	149 (7.1)
Handshakes or hugs?	1698 (81.2)	252 (12.1)	140 (6.7)
Touching an item someone else touched?	1518 (72.6)	376 (18.0)	196 (9.4)
Sharing and eating from the same dish?	1774 (84.9)	193 (9.2)	123 (5.9)
**Attitudes:**			
Has your daily life been disturbed (interrupted, changed) by COVID-19?	1128 (54.0)	962 (46.0)	· ·
Do you worry about contracting COVID-19?	1679 (80.3)	411 (19.7)	· ·
**Practices:**			
Because of COVID-19:			
Do you avoid crowded places?	1314 (62.9)	752 (36.0)	24 (1.1)
Do you wash your hands more frequently?	1879 (89.9)	196 (9.4)	15 (0.7)
Do you have access (can you buy or receive) to alcohol, hand sanitizer? (or have you bought more recently?)	1484 (71.0)	577 (27.6)	29 (1.4)
Do you keep a distance from people with influenza-like symptoms (flu/colds)?	1378 (65.9)	696 (33.3)	16 (0.8)
Do you wear a face mask?	585 (28.0)	1479 (70.8)	26 (1.2)

*The sample size for this question was N = 2224. Only the 2090 individuals who responded 'Yes' were asked to complete the subsequent knowledge, attitudes, and practices (KAP) questions.

In terms of knowledge surrounding potential preventive measures to protect people from infection, handwashing was identified by 1719 respondents (82.2%) while the remaining options were chosen by less than half of the respondents (**Table 4**). Social distancing and avoiding crowds were recognized as preventive measures by 677 (32.4%) and 849 (40.6%) respondents, respectively. Some problematic options were selected: 66 (3.2%) people identified drinking alcohol and 24 (1.1%) identified carrying a ginger pouch as potential preventive measures. Higher levels of education were associated with greater knowledge of COVID-19 transmission routes but not of proper preventive measures against the virus, but having access to at least one phone was associated with greater knowledge of both (**Table 5**).

**Table 4:** Knowledge, attitudes and practices toward COVID-19 among income-poor households in the Philippines (continued)

Question (n = 2090)	No.	%
**Knowledge:**		
How can you protect yourself against COVID-19?:		
Hand washing	1719	82.2
Face masks	1024	49.0
Hand sanitizer	927	44.4
Social distancing (staying away from people who are sick)	677	32.4
Vitamins, Calamansi tea or other citrus fruit, herbal remedies	683	32.7
Avoiding large crowds	849	40.6
Drinking alcohol	66	3.2
Changing clothes often or after being in public	225	10.8
Ginger pouch	24	1.1
Other	72	3.4
**Attitudes:**		
If you have symptoms like fever, cough, and sore through, what would you do? (select all that apply):		
Stay at home and wait to get better	762	36.5
Use stored medicine at home	724	34.6
Contact *barangay* health worker (community health worker)	1033	49.4
Seek antibiotics	423	20.2
Visit RHU (Rural Health Unit)	720	34.4
Visit a pharmacy	309	14.8
Visit a public hospital	911	43.6
Visit a private hospital	252	12.1
See the local hilot (traditional medicine)	145	6.9
Other	46	2.2
I don’t know	15	0.7

**Table 5:** Groupwise means and results of Kruskal-Wallis test on demographic and social capital determinants of COVID-19 knowledge and practices among income-poor households in the Philippines

Variables (n = 2090)	Number of COVID-19 transmission modes identified				Number of COVID-19 preventive measures identified				Number of COVID-19 preventive measures taken				
**Mean**	**SD**	**χ^2^**	***P*-value**	**Mean**	**SD**	**χ^2^**	***P*-value**	**Mean**	**SD**	**χ^2^**	***P*-value**
**Income:**			0.55	0.76			1.39	0.50			2.13	0.34
Less than US$ 1.00	4.11	1.43			2.49	1.35			3.15	1.44		
US$ 1.00-US$ 2.00	4.13	1.41			2.53	1.29			3.21	1.45		
Greater than US$ 2.00	4.09	1.38			2.53	1.21			3.25	1.47		
**Geotype:**			7.44	0.02			20.54	<0.001			23.04	<0.001
Urban	3.95	1.40			2.11	1.11			3.34	1.23		
Rural	4.12	1.42			2.54	1.32			3.09	1.48		
Coastal	4.15	1.45			2.60	1.37			3.52	1.38		
**Education:**			14.33	0.01			6.12	0.29			16.95	<0.01
No Education	3.33	1.88			2.08	1.18			2.69	1.60		
Elementary	4.01	1.53			2.52	1.34			3.07	1.50		
Partial High School	4.23	1.31			2.51	1.31			3.23	1.43		
High School Graduate or ALS	4.10	1.33			2.54	1.21			3.46	1.28		
Partial College or Vocational School	4.23	1.21			2.41	1.32			3.09	1.46		
Complete College or Higher	4.24	1.45			2.74	1.40			3.52	1.09		
**Has at least 1 phone:**			5.02	0.03			14.38	<0.001			28.80	<0.001
No	3.95	1.55			2.30	1.27			2.83	1.50		
Yes	4.15	1.38			2.56	1.32			3.26	1.42		
**Has a working TV:**			1.13	0.29			0.00	0.96			9.55	<0.01
No	4.07	1.45			2.52	1.32			3.08	1.46		
Yes	4.16	1.38			2.50	1.31			3.27	1.42		
**Trusts neighbor:**			11.39	0.02			29.48	<0.001			8.06	0.09
No trust	4.29	1.24			2.14	1.16			3.46	1.24		
Tentatively trust	3.95	1.47			2.44	1.31			3.18	1.40		
Neutral	4.15	1.40			2.64	1.38			3.23	1.41		
Moderately trust	4.16	1.42			2.55	1.26			3.19	1.44		
Very trusting	4.00	1.45			2.20	1.25			2.94	1.60		
**Trusts Barangay Health Station or RHU:**			5.85	0.21			37.33	<0.001			8.86	0.06
No trust	3.82	1.33			2.18	1.25			3.36	1.43		
Tentatively trust	4.01	1.40			2.71	1.28			3.59	1.11		
Neutral	4.12	1.43			2.75	1.41			3.24	1.44		
Moderately trust	4.15	1.40			2.49	1.25			3.15	1.46		
Very trusting	4.05	1.45			2.25	1.28			3.08	1.48		
**Knows the Barangay Captain:**			1.37	0.24			0.56	0.45			0.56	0.46
No	4.19	1.47			2.42	1.27			3.13	1.31		
Yes	4.11	1.42			2.51	1.32			3.18	1.45		

SD – standard deviation, RHU – rural health unit, ALS – alternative learning system

### COVID-19 attitudes

To examine health-seeking intentions, respondents were asked what they would do if they exhibited symptoms like a fever, cough or sore throat (**Table 4**). Overall, 1033 (49.4%) said they would contact a *barangay* health worker (ie, community health worker) or seek health advice elsewhere: there were 911 (43.6%) respondents that indicated they would visit a public hospital and 720 (34.4%) respondents who would consider visiting a rural health unit (RHU). Respondents also considered handling the symptoms themselves with 762 (36.5%) who reported the intention to stay at home and wait to get better and 724 (34.6%) respondents who selected the option to use medicines they may have at home.

At the time of the survey, 1128 respondents (54.0%) reported that their life had been disturbed by COVID-19, but 1679 (80.3%) indicated that they were worried about contracting the virus, suggesting that people were responding in alarm to the information that was being circulated about the disease. This response is reflected in the logistic regression model (**Table 6**) showing that the odds of being worried about contracting the virus for those who had a television was 1.31 times (95% confidence interval (CI): [1.03, 1.66]) that of respondents who did not have a television. For each mode of transmission that a respondent was aware of, they were also more likely to be worried about contracting the virus (odds ratio (OR) = 1.13; 95% confidence interval (CI) = 1.05-1.22) (**Table 7**). Merely consuming information about COVID-19 appears to have generated concern, but those who reported having their daily life disturbed by COVID-19 were *much* more likely to report being worried about contracting the virus (OR = 7.33; 95% CI = 5.51-9.75).

**Table 6:** Results of logistic regression of demographic and social capital determinants of COVID-19 risk perception attitudes among income-poor households in the Philippines

Variables (n = 2070)	Worried about contracting the virus	
**Adjusted OR (95% CI)**	***P*-value**
**Income:**		
Less than US$ 1.00	(ref)	
US$ 1.00 – US$ 2.00	0.73 (0.57-0.93)	0.01
Greater than US$ 2.00	0.81 (0.56-1.18)	0.27
**Geotype:**		
Urban	(ref)	
Rural	1.01 (0.67-1.53)	0.95
Coastal	1.09 (0.66-1.80)	0.73
**Education:**		
No Education	(ref)	
Elementary	2.12 (1.02-4.38)	0.04
Partial High School	1.9 (0.91-3.99)	0.09
High School Graduate or ALS	2.22 (0.99-5.01)	0.05
Partial College or Vocational School	1.84 (0.80-4.21)	0.15
Complete College or Higher	1.94 (0.69-5.46)	0.21
**Has at least 1 phone**	1.27 (0.94-1.70)	0.12
**Has a working TV**	1.31 (1.03-1.66)	0.03
**Trusts neighbor:**		
No trust	(ref)	
Tentatively trust	1.18 (0.54-2.61)	0.68
Neutral	0.66 (0.32-1.35)	0.25
Moderately trust	0.95 (0.46-1.95)	0.89
Very trusting	0.72 (0.3 -1.54)	0.40
**Trusts Barangay Health Station or RHU:**		
No trust	(ref)	
Tentatively trust	1.86 (0.34-10.04)	0.47
Neutral	1.45 (0.30-7.10)	0.65
Moderately trust	1.38 (0.28-6.75)	0.69
Very trusting	1.34 (0.27-6.60)	0.72
**Knows the Barangay Captain:**	0.64 (0.34-1.19)	0.15

OR – odds ratio, RHU – rural health unit

**Table 7:** Results of logistic regression of knowledge, attitudes and practices determinants of COVID-19 risk perception attitudes among income-poor households in the Philippines

Variables (n = 2070)	Worried about contracting the virus	
**Adjusted OR (95% CI)**	***P*-value**
Know the main modes of transmission	1.15 (1.07-1.24)	<0.01
Has your daily life been disturbed (interrupted, changed) by the new coronavirus?	7.33 (5.519.75)	<0.001

OR – odds ratio, CI – confidence interval

### COVID-19 practices

Handwashing appears to be the most common preventive practice, adopted by 1879 (89.9%) respondents in response to COVID-19. Interestingly, there were discrepancies observed between the proportion of participants who reported adopting certain practices and the proportion of participants who identified those same practices as a potential preventive measure. While 1314 (62.9%) respondents reported that they now avoided crowded places because of COVID-19, only 849 (40.6%) selected ‘avoiding large crowds’ as a preventive measure against the virus. Similarly, 1378 (65.9%) respondents said that they currently keep a distance from people with influenza-like symptoms, but when framed as a potential preventive measure, only 677 (32.4%) of respondents selected ‘social distancing’ as an option. Finally, only 585 (28.0%) reported wearing face masks as a response to the virus. Factors associated with the adoption of preventive practices include having higher educational qualifications and having at least one working phone (**Table 5**).

Results from **Table 8** indicate that more preventive measures are taken by those with better knowledge of modes of transmission, those who report having their daily lives disturbed by the virus, and those who are worried about contracting COVID-19.

**Table 8:** Groupwise means and results of Kruskal-Wallis Test on knowledge, attitudes and practices determinants of COVID-19 practices among income-poor households in the Philippines

Variables (n = 2090)	Number of COVID-19 preventive measures taken			
**Mean**	**SD**	**X^2^**	**I-value**
**Know the main modes of transmission:**			187.19	<0.001
None	1.62	1.56		
One	2.31	1.58		
Two	2.59	1.45		
Three	2.81	1.36		
Four	3.24	1.34		
Five	3.44	1.35		
**Has your daily life been disturbed (interrupted, changed) by the new coronavirus?:**			163.53	<0.001
No	2.73	1.51		
Yes	3.56	1.27		
**Do you worry about contracting the new coronavirus?:**			203.75	<0.001
No	2.23	1.58		
Yes, a little	3.27	1.40		
Yes, very much	3.61	1.16		

## DISCUSSION

COVID-19 is a global health emergency, and in settings with fragile health systems and widespread misinformation about the virus, many issues that have been observed over the course of the pandemic are likely to be exacerbated. This study provides important and timely insights into how households from the lowest income quintile of a LMIC receive and understand information regarding a novel emerging disease [33]. At the time the survey was administered, a large majority (94.0%) of respondents had already heard of COVID-19. We found that even during the earlier stages of the pandemic, people already perceived the spread of the virus as a cause for concern that could impact them directly. Only half (54.0%) of all respondents reported that their daily life had been disrupted by COVID-19, but 80.3% were worried about contracting the virus, suggesting an initial response of panic due to the information that was being circulated. However, although knowledge of transmission routes was high, appropriate preventive measures against COVID-19 were not well-identified. The majority (82.2%) of respondents recognized hand hygiene as an important preventive measure against infection, but there was a lack of identification of other key measures such as social distancing and avoiding large crowds, and despite an association between knowledge and practice, the proportion of people adopting preventive practices was relatively low.

Similar results were reported in KAP studies in low-income settings for other vector-borne and infectious diseases, such as malaria, tuberculosis, and Influenza A (H1N1). Awareness of the diseases were generally high [34,35], and a positive association between knowledge and preventive practices adopted was consistently demonstrated across all studies [34-37]. However, a KAP study conducted in a community living along the Thai-Myanmar border that was characterized as the least likely to receive health education from the government showed that knowledge can be inequitably distributed. The study population displayed a lack of knowledge about H1N1 with regards to disease transmission, common symptoms and self-protection practices [37], reinforcing the importance of targeted health education. There have also been a number of studies that have explored KAPs of COVID-19, but many were exclusively targeted toward health care workers [18,20,21,24]. Those that aimed to survey the general population have only been conducted in China or the United States [19,22,23,38], and these studies have acknowledged the need to further understand the KAPs of vulnerable populations that may have lower health literacy.

Although the respondents in this study are not representative of the general public, they represent a subset of the population that has limited access to health services and are often marginalized from social services [39-42]. In the Philippines, health care utilization differs substantially based on socioeconomic position: in 2017, only 59.7% of those in the lowest wealth quintile had any health insurance, compared to 83.2% among those in the highest wealth quintile [43]. Low-income populations in the Philippines also disproportionately suffer from undernutrition, and in combination with lack of access to health care, we see rates of child mortality that are six times higher in the lowest wealth quintile, compared to the wealthiest [43]. During health crises and emergencies, these populations can be overlooked and deprioritized, which is why understanding their KAPs during the earlier stages of an emerging pandemic can help government departments, multi-lateral organizations, and NGOs direct public health response and communication strategies appropriately [44].

It is notable that, by far, most of this population reported obtaining their information through traditional media sources, such as television and radio, as opposed to social media. In addition to limited internet connectivity in many areas in the Philippines [45], many people may not own devices that can access social media due to the demographic represented in this study. This finding highlights the need for public health communication strategies to avoid a singular focus on social and digital media as mediums for information dissemination.

An important knowledge gap was observed regarding proper preventive measures against COVID-19. Compared to potential transmission routes, the proportion of people that identified appropriate ways to protect themselves was low, and there was also a disconnect between preventive practices *identified* by respondents and those they report to have *adopted*. Some respondents reported social distancing and avoiding large crowds in response to the virus, despite not having selected them as preventive measures in the survey. This suggests that while public health messaging may have been able to emphasize the importance of adopting certain practices, the rationale behind these preventive measures has not been well-communicated to these populations. Alternatively, people may be practicing certain measures but are unsure of the effectiveness of these measures for disease prevention. With the exception of handwashing, the relatively low proportion of people adopting preventive practices demonstrates a need to increase or improve public health knowledge translation in outbreak scenarios in contexts that may not have regular access to information, as well as to explore other potential barriers to uptake that may exist among low-income populations.

The findings of this study also has implications for the development of response initiatives in low-income settings. Social distancing and avoiding large crowds are examples of non-pharmaceutical interventions (NPIs), which aim to control transmission by reducing contact rates in the general population. Until a vaccine becomes available, such measures will continue to be vital in the global response against COVID-19 [46], but are particularly important in this population where any novel pharmaceutical intervention may be inaccessible due to cost or poor distribution. While these data highlight a lack of understanding of NPI strategies among households experiencing extreme poverty, there is also a growing recognition that people living in densely populated urban slums and other income-poor communities are unlikely to have the spatial and economic capacity to practice social distancing and self-isolation [1,47]. Responses such as cash transfers to enable households to “stay-at-home” have been suggested [2], as mitigation strategies will need to account for populations experiencing poverty that may not have fixed incomes [47,48]. It is encouraging, however, that respondents with more knowledge of potential transmission routes also reported taking a greater number of preventive measures against the virus. While this association between knowledge and practice reinforces the importance of widespread public health messaging and information campaigns as the situation continues to develop, in low-income settings, there is a need for *targeted* messaging and health education that carefully considers the difficulties these populations may face in attempting to practice NPIs.

With regards to the planning of primary care response initiatives, it is particularly important to highlight the observed lack of intention to access health services even if people were to exhibit symptoms of COVID-19. Inequitable health care utilization is already observed in this population, even during times without public health emergencies, due to barriers such as long distances to health services, losing a day of income for travel, the affordability of services, and facing the stigma associated with potential diagnoses of infectious diseases [41,49,50]. These findings indicate that such barriers to seeking health care may also be pertinent to COVID-19 care if or when it becomes needed in these households. While strengthening the capacity of health facilities and health care workers represents a critical and much need response to the COVID-19 pandemic, these responses must also consider the longstanding structural barriers that populations experiencing poverty face in accessing and using the health system in LMICs.

Some limitations of this study must be acknowledged. Given this was a rapid survey, we had to rely on self-reported, instead of observed, practices, and we are unable to verify whether this measure was affected by social desirability bias. Also, as the severity of the pandemic increased, public health campaigns have likewise intensified in the Philippines since the survey was conducted, so the results of this study may not reflect current KAPs. While it would be informative to repeat this survey during this heightened phase of the virus outbreak, it is not feasible to survey this population online due to their limited access to devices with internet access. In-person follow-up surveys are also not possible due to ongoing travel restrictions and social distancing guidelines.

## CONCLUSIONS

This study examined KAP on COVID-19 among households experiencing extreme poverty in the Philippines during the earlier stages of the pandemic. In the context of a fragile health system and the spread of misinformation concerning COVID-19, it is important to understand how populations that have limited access to health services and information perceive this issue, and in particular, appropriate responses or preventive measures. This population showed high degree of knowledge of transmission routes, but with the exception of handwashing, they had limited knowledge in the identification and adoption of other preventive practices. Those who identified a greater number of transmission modes also adopted more preventive practices. This association between knowledge and practices demonstrates the importance of prompt and accurate public health communication. As many COVID-19 response strategies employed by high-income countries are unlikely to be feasible in LMIC settings, targeted health education and tailored guidance for public health response must be developed with careful consideration of extreme low-income households.

## Additional material

Online Supplementary Document
