# Supplementary material for: Knowledge, attitudes and practices of COVID-19 among income-poor households in the Philippines: A cross-sectional study
Source: J Glob Health. 2020 Jun 11;10(1):011007. doi: 10.7189/jogh.10.011007 (PMC7294392; doi:10.7189/jogh.10.011007)
Supplement: Online Supplementary Document [file jogh-10-011007-s001.pdf]

Supplementary online material

Knowledge, Attitudes and Practices (KAP) of the novel Coronavirus 2019 (COVID-19) among Income Poor Households in the Philippines: A cross-sectional study

CONTENTS

1 ICM 2019 Survey Covid-19 Questions
2 Table S1. Description of geographical area types where survey respondents resided
3 Note on exchange rate

Appendix S1. ICM 2019 Survey Covid-19 Questions

kn_0 Have you heard of the new coronavirus (Covid-19)?
1 Yes
0 No
-98 I don’t know

kn_1 What do you think? Can the new coronavirus be transmitted (caught or spread) by…?:

kn_1a Cough and sneeze
1 Yes
0 No
-98 I don’t know

kn_1b Face to face talking
1 Yes
0 No
-98 I don’t know

kn_1c Handshakes or hugs
1 Yes
0 No
-98 I don’t know

kn_1d Indirect hand contact (touching an item someone else touched)
1 Yes
0 No
-98 I don’t know

kn_1e Food (sharing and eating from the same dish)
1 Yes
0 No
-98 I don’t know

kn_2 If you have symptoms like fever, cough, and sore throat, what would you do?
Select all that are appropriate.

1 Stay at home and wait to get better
2 Use stored medicine at home
3 Contact *barangay* health worker
4 Seek antibiotics
5 Visit rural health unit (RHU)
6 Visit a pharmacy
7 Visit public hospital
8 Visit private hospital
9 Local hilot (traditional medicine)
-66 Other *(please specify)*
-98 I don't know

att_1 Has your daily life been disturbed (interrupted, changed) by the new coronavirus?
Has anything changed in your life since hearing about the new coronavirus?

1 Yes
0 No

att_2 Do you worry about contracting the new coronavirus?

0 No
1 Yes, a little
2 Yes, very much

pr_0 Since the news of the coronavirus…

pr_1 Do you avoid crowded places?

1 Yes
0 No
-98 I don't know

pr_2 Do you wash your hands more frequently?

1 Yes
0 No
-98 I don't know

pr_3 Do you have access (can you buy or receive) to alcohol, hand sanitizer? (or have you bought more recently?)

1 Yes
0 No
-98 I don't know

pr_4 Do you wear a face mask now because of the new coronavirus?

1 Yes
0 No

-98 I don't know

pr_4a (IF pr_4 = YES) What type of mask(s) do you wear?
Select all that are appropriate.
1 Motorcycle mask
2 Bandana
3 Surgical
4 N95
-66 Other (please specify)

pr_4b (IF pr_4 = NO) If no, is it because…
Select all that are appropriate.
1 All are out of stock
2 They are too expensive
3 I gave it to someone in my family
4 I don't think it helps against coronavirus
-66 Other *(please specify)*

pr_5 Do you keep a distance from people with influenza-like symptoms (flu/colds)?
Ex: people coughing, sneezing, with fever, with runny nose, etc.
1 Yes
0 No
-98 I don't know

inf_1 Where did you learn and stay up to date about the new coronavirus?
Select all that are appropriate.
1 Television (news)
2 Radio
3 Social media (Facebook, Instagram, etc.)
4 Internet (websites, blogs, etc.)
5 Friends, relatives, and/or neighbours
6 Government officials (local government officials, *barangay* health workers, etc.)
7 Announcements at work
-66 Other *(please specify)*

kn_3 How can you protect yourself against the new coronavirus?
Select all that are appropriate.
1 Hand washing
2 Face masks
3 Hand sanitizer
4 Social distancing (staying away from people who are sick)
5 Vitamins, Calamansi tea or other citrus fruit, herbal remedies
6 Avoiding large crowds
7 Drinking alcohol
8 Changing clothes often or after being in public
9 Ginger pouch

-66 Other *(please specify)*

2 Table S1

Table S1. Description of geographical area types where survey respondents resided

Geographical area type	Description
Rural	Rural communities are predominantly engaged in the agricultural industry, and households are relatively more spread out.
Coastal	The proximity of households in coastal communities are a mixture of dense and semi-dense housing, and the main industry is fishing or collecting shells.
Urban	Urban communities are located within or in the vicinity of city centres and household density is higher than in rural settings.

3 Note on exchange rate

The PHP/USD exchange rate was taken on April 27, 2020 (1 PHP = 0.019755 USD).
